# Contributions to Clinical Medicine

**Published:** 1899-09

**Authors:** 


**Contributions to Clinical Medicine.** By T. M'CALL ANDERSON, M.D. Edited by JAMES HINSHELWOOD, M.D. Pp. x., 415. Edinburgh: Young J. Pentland. 1898.

One who has been a hospital physician and clinical teacher for upwards of a quarter of a century must always have something worth relating. This volume will be specially welcome to Dr. M'Call Anderson's old students. Some of the papers are a little scrappy, and some rather old; but they are of world-wide interest, and they are all presented in good form, well printed on thick paper, easy to read, and well worthy of any time which may be spent upon them.

